# Social Media Contexts Moderate Perceptions of Animals

**DOI:** 10.3390/ani10050845

**Published:** 2020-05-14

**Authors:** Elizabeth Riddle, Jill R. D. MacKay

**Affiliations:** Royal (Dick) School of Veterinary Studies, The University of Edinburgh, Scotland EH25 9RG, UK; riddleel14@gmail.com

**Keywords:** digital cultures, captive primates, exotic pets, digital human-animal interactions

## Abstract

**Simple Summary:**

Social media sites may contribute to the changing ways we see animals. On these sites, people can present animals in different contexts, depending on what message they want to convey, and this may change how people perceive animals, for example making people more likely to want an exotic species as a pet. We showed a mock-up site to 211 people. All people were shown the same image of a primate, but half were shown a negative story and half were shown a positive story. People shown the negative story thought that the primate was more stressed. People responded cautiously to the social media site, even when they thought the primate was stressed. We conclude that social media may not be an honest representation of how people think about primates.

**Abstract:**

The rapid rise of social media in the past decade represents a new space where animals are represented in human society, and this may influence human perceptions, for example driving desire for exotic pet keeping. In this study, 211 participants (49% female) between the ages of 18 to 44 were recruited to an online survey where they viewed mock-up pages from a social media site. All participants saw the same image of a primate but were randomly assigned to a pro exotic pet keeping or anti exotic pet keeping narrative condition. When participants were presented with the anti narrative they perceived the animal to be more stressed (χ^2^ = 13.99, *p* < 0.001). In free text comments, participants expressed reservations in the face of a narrative they disagreed with in free text comments. Overall, this study found evidence to suggest that people moderate their discussions on human-animal interactions based on the social network they are in, but these relationships are complex and require further research.

## 1. Introduction

Social media sites (SMS) are a rapidly expanding form of human communication. They can be defined as “virtual places that cater to a specific population in which people of similar interest gather to communicate, share, and discuss ideas” [1] p. 169. Popular sites, especially among teenagers, include Facebook, Twitter, Instagram, YouTube, and Tumblr [2]. In the US, Facebook is the most visited SMS [3] and claimed a global reach of 2 billion users in 2017 [4]. In 2012 over half of adults under 54 and 86% of adults aged 18–29 used SMS, compared to less than 10% of the population in 2005 [5]. It has been proposed that this shift in human communication created a digital culture, a unique method of sharing social norms and curating behaviors [6]. This culture can have both positive and negative impacts, e.g., as women athletes become more visible they can act both as a role model online, but also receive online abuse [7]. A modern, cohesive definition of culture from a sociology point of view is difficult to find. However studies of culture need to recognize both the unique space in which the culture exists in and the performative aspect of culture, i.e., its ability to be shared [8]. One suggestion [9] is that researchers ought to explore differences between psychosocial effects and platform-driven behavior, as platforms bring their own ecosystems and cultures to the data collected. For example, one study found that ‘social gratification’, the number of ‘likes’, ‘comments’, and ‘shares’ a post received, are a positive driver of sharing activity on Facebook [10]. This is unsurprising, given human behavior is strongly mediated by social reputation [11], and SMS interactions provide a mechanism for people to judge their community contributions.

As humans use online spaces to record their relationships with other humans, they also use them to describe and contextualize their relationships with non-human animals (hereafter ‘animals’). Human-animal interactions (HAI) covers the gamut of experiences humans have had with animals in all forms of cultures and societies, from animal worship to animal use and animal companionship [12]. Traditional media shapes, and is shaped by, HAI. In experimental settings, participants who viewed television advertisements featuring non-human primates (hereafter ‘primates’) in entertainment contexts, for example, seeing a chimpanzee interact with an object like a human would, had an increased likelihood of agreeing that chimpanzees should be owned as pets [13]. Similarly, when participants in a survey viewed images of chimpanzees in proximity to humans [14], and anthropomorphic still images of chimpanzees [15], they perceived chimpanzees to be more suitable pets. Many of the relationships observed between traditional media and HAI can also be observed in digital culture. Animals are often considered ‘totemic’, representing some aspect of a person or society that can be used as shorthand for communication [16], such as the animal ‘meme’ [17]. In one case, an image of a Malayan sun bear progressed from a classic ‘meme’ example of absurdist humor to an outlet for confessing socially taboo topics [18]. This style of HAI is entirely one-sided, with humans appropriating animals and possibly sublimating animal needs in favor of their own. For example, the popularity of a video of a slow loris being ‘tickled’ was associated with a number of users expressing a desire to interact with the animals as pets, despite their at-risk conservation status [19]. From an ethological perspective, the behaviors displayed during ‘tickling’ are indicative of stress and poor welfare [20].

In sociology, the content of written media and the attitudes express within can give an insight into how society views certain issues, and the internet allows individuals to editorialize their own important issues, i.e., animal obituaries [21]. This can be viewed with the lens of ‘framing’ theory, which suggests that how information is presented, e.g., its qualitative of emotional presentation, changes the way that information is perceived by the recipient [22]. Within SMS, framing is used to recruit support for a given cause, often with activism contexts [23]. For example, activists may present information in a certain way to mobilize supporters, through bridging frames (e.g., presenting two similar arguments), amplifying frames (e.g., providing more information to support an idea), extending frames (e.g., through reaching more individuals for greater impact), and frame transformation (e.g., redefining a message in light of a new frame) [24]. For example, discussion of ‘positive animal welfare’ has an influence on key stakeholders, such as farmers, in supporting positive animal welfare changes [25]. Online animal activism is often highly emotionally charged and may be suited to framing studies; however, these presentations also encourage strong disagreement from some with contrasting opinions, which devalues the message being delivered [26,27]. It is not clear how framing impacts attitudes to animals in SMS.

In this study, we sought to explore how the framing of a particular SMS may affect the users’ attitudes towards exotic pets. The primary hypothesis was that users exposed to pro exotic pet content would be more accepting of exotic pets than those exposed to anti exotic pet content. The secondary hypothesis was that these attitudes would be more strongly expressed when the content had a high ‘social loading’, e.g., had received many ‘likes’.

## 2. Materials and Methods

### 2.1. Ethical Review

This study was reviewed and approved by Human Ethical Review Committee within the Royal (Dick) School of Veterinary Studies (HERC_20_16).

### 2.2. Choice of Social Media Site

An ‘access control scheme’ site was considered most appropriate as these are commonly used by people ‘researching’ purchasing decisions [28,29]. ‘Access control scheme’ sites, such as Facebook, allow users to select who to share content with and work primarily through their network (friends, family, ‘liked’ pages or groups [30,31]) but do allow for unknown users to interact with one another.

Facebook allows for the creation of ‘groups’ which are ‘followed’ by individual users. In order to produce a mock-up Facebook group which would be pro or anti exotic pet keeping, a variety of terms relating to exotic pet ownership were used with Facebook’s inbuilt search function. These terms were “funny animals”, “exotic pets”, “monkey”, “monkid”, “monkey pet”, and “monkey baby”. Popular results consisted of pro and anti exotic pet pages and groups, personal posts about exotic pets, and short videos of exotic animals. Given the focus of the search terms, it is unsurprising that most animals featured were primates, however large cat species such as tigers, cheetahs and servals were also observed. It should be noted however that we were not interested in species identification, but rather the general topics of the posts and comments in order to recreate believable pages. Pages were rarely species specific, and outside of easily recognizable animals such as tigers, posts rarely identified the specific species. Most primates were referred to as ‘monkeys’. Given that users have not consented for this data to be used in research, this study opted instead to create a survey with a mock Facebook page in order to explore users’ self-reports of behavior, in line with the Association for Internet Researcher’s ethical guidance [32]. In order to produce fake pages, we categorized the informational elements of a typical Facebook group post as follows: ‘content’ was the media or text being shared, ‘commentary’ was the original poster’s editorializing of that content, ‘social loading’ was the quantity of interactions, e.g., ‘likes’ and ‘shares’ that the content received, and finally, the ‘social network response’ was the user’s discussion of the content (see study design below).

### 2.3. Participants

Participants were recruited to the study via the commercial online survey platform SurveyMonkey’s (www.surveymonkey.com) volunteer respondent cohort. This cohort provides demographic data to the platform and can be targeted for wide scale recruitment. The volunteers are incentivized by SurveyMonkey to complete surveys by a small (approx. $1) donation to one of the SurveyMonkey affiliated charities. It is not known what charities were selected by participants or how many were utilizing the incentivisation. Using a purchased survey cohort to collect responses allowed us to recruit from the general public and avoid recruiting people via university channels, as followers of animal-related organisations on SMS would have done so because they are presumably interested in animal welfare. The selected demographic contained adults aged 18–44 that resided in the United States. Participants who matched the criteria were emailed the link by the commercial platform automatically until the minimum purchase threshold of 200 respondents was reached, meaning participants received no information about the survey in their initial email. In total, we received 238 returned surveys. Responses containing incomplete surveys were discarded, resulting in 211 useable responses. Due to concerns from the ethics panel regarding collecting unnecessary identifying data, the age category was retained in the platform’s demographic data and was not asked for in the survey specifically. There were no significant differences in gender, age, or educational status across the four conditions (Table 1).

### 2.4. Study Design

Survey participants were shown a mock-up image of a Facebook group page (condition). There were four conditions: pro exotic pet keeping with high social loading (Pro-High), pro exotic pet keeping with low social loading (Pro-Low), anti exotic pet keeping with high social loading (Anti-High), and anti exotic pet keeping with low social loading (Anti-Low). For all four conditions, the content was the same animated image of a cotton-top tamarin (*Saguinus oedipus*). This image and individual was used for convenience and because the authors’ owned the rights to the video. The colour image depicts the tamarin standing on an artificial branch, looking at the surroundings. The background is the enclosure wall, painted different shades of green. A rope and artificial branch are the only furnishings in frame.

We observed groups most commonly shared and discussed video content. We opted for a moving image due to the technological industry’s comment on SMS as places that encourage video media consumption [33]. Therefore, we decided to produce a moving or animated image. Due to technological limitations at the time, we were unable to embed a video into the survey, and so a GIF (graphics interchange format) was created from the video to create a looped animation which would play like a video and be robust across different devices that may access the survey.

**Figure 1:**
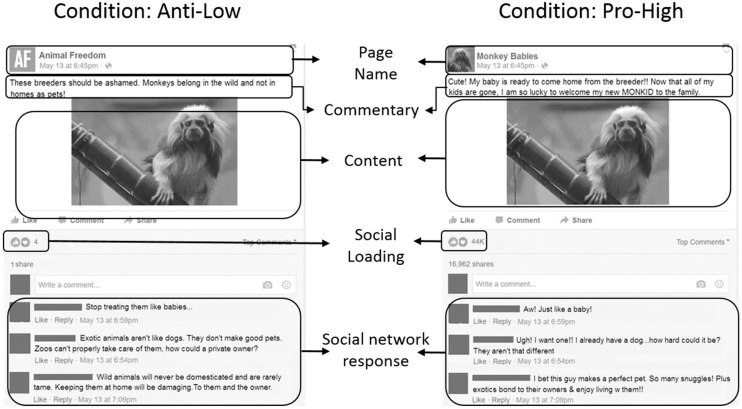
Comparison of two fictional Facebook groups, the ‘Anti-Low’ condition and the ‘Pro-High’ condition. Note that the ‘Anti-High’ condition is identical to the ‘Anti-Low’, aside from the number of reactions, and vice versa for ‘Pro-Low’ and ‘Pro-High’.

Both pro and anti narratives featured the same pro or anti content respectively. The themes expressed in the social network responses were similar in content but different in valence between pro and anti narratives. Both high conditions stated the page had received 44 K + ‘likes’, while both low conditions featured 4 ‘likes’ (Figure 1). A few months prior to data collection, Facebook had introduced ‘reactions’ as well as ‘likes’ [34]. We decided to include ‘like’ and ‘love’ as the reactions as we judged a post using only ‘likes’ would appear immediately dated, but there was no distinction between how many people ‘liked’ versus ‘loved’ each post, similar to Facebook’s presentation at the time. The comments were rewritten from real comments observed on SMS, in order to express similar themes with different emotional valence in each narrative. For example, the comment about the primate being ‘like a dog’ was paraphrased from recurring observations online (Table 2). Each participant only saw one condition, which they were assigned via their provided birth month to ensure approximate equal numbers across conditions as there was no facility for randomising condition entry in the platform available.

After being shown the image of the Facebook group, participants answered a series of questions regarding their attitudes towards the animal and the commenters in the image (Table 3). Even (four-point, Strongly Disagree, Disagree, Agree, Strongly Agree) Likert scales were used to obtain a forced decision on whether the environment was appropriate for the primate and whether the primate would make a suitable pet. Mid-points on odd Likert-like scales can be undesirable where there is concern that respondents may conceal answers they perceive to be socially unacceptable [35].

### 2.5. De-Brief

To avoid participants leaving with an altered view of this subject, a de-brief was given at the end of the survey. The final page showed a ‘negative-high’ image, ‘positive-low’ image, and the video which the GIF was created from. It also stated that the purpose of the research was to study the relationship between SMS use and human perceptions of non-human animal welfare. It was additionally requested that participants did not share any information about the survey on SMS or any other media platform, although this was not followed up by the researchers.

### 2.6. Analysis

The three Likert scale questions, the suitability of the primate as a pet, the suitability of the environment, and the knowledge of the original poster, were compared across SMS context, age, gender, and education via Kruskal-Wallis tests using R Version 3.6.0 (“Planting of a Tree”, R Core Team, 2019) and the ‘likert’ package [36]. Kruskal-Wallis tests were interpreted through one- and two-tailed multiple comparison tests to establish which groups showed significant differences with the use of the pgirmess package [37].

After seeing the page and ensuing discussion, participants were asked what they would write if they were to respond to the discussion. Both authors contributed to a thematic analysis identifying the broad themes present in the comments and then JM coded the themes via qualitative data management software (N Vivo 11, QSR International). During coding, JM was blind to the condition the participant was in and used a constructive grounded theory method [38] with the fundamental question being ‘how do participants resolve the animal welfare issues presented in the narrative in their own comments’. To explore differences between demographics and treatments, a series of χ^2^ analyses were run.

## 3. Results

### 3.1. Participant Attitudes to Captive Primates

**Figure 2:**
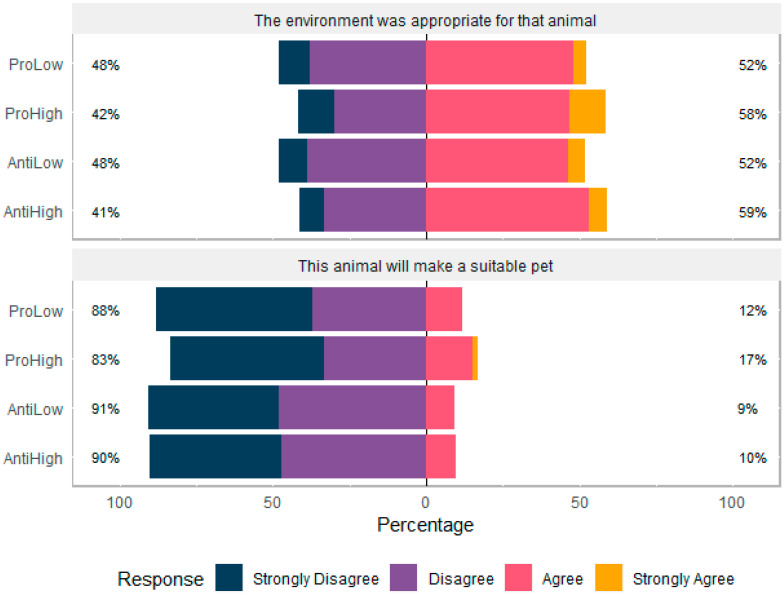
Participants agreement rating regarding environment and pet suitability of primate across social media site (SMS) condition (N = 211).

SMS context (pro versus anti) had no effect on whether participants thought the environment was appropriate for the animal (H = 1.1549, df = 3, *p* = 0.7638) or whether the animal would make a suitable pet (H = 0.4435, df = 3, *p* = 0.9311, Figure 2). Age, gender, and education had no effect on these scores in multiple comparison tests. A little over half of all participants (55.2%) felt that the environment the primate was pictured in was suitable. Across all conditions, only 11.4% of participants felt the animal would make a suitable pet and the majority (74.9%) stated that they personally would not like the primate as a pet. Participants were asked about the animal’s mood, and despite being presented with the same image, participants’ responses differed across experimental condition. Participants who were shown the pro narrative were more likely to agree that the primate was stressed (χ^2^ (1, N = 211) = 13.99, *p* < 0.001, OR = 2.9), whereas those who were shown the anti narrative were more likely to respond ‘don’t know’ (χ^2^ (1, N = 211) = 10.21, *p* =0.001, OR = 2.8).

### 3.2. Participant Attitudes to Original Poster of Content

**Figure 3:**
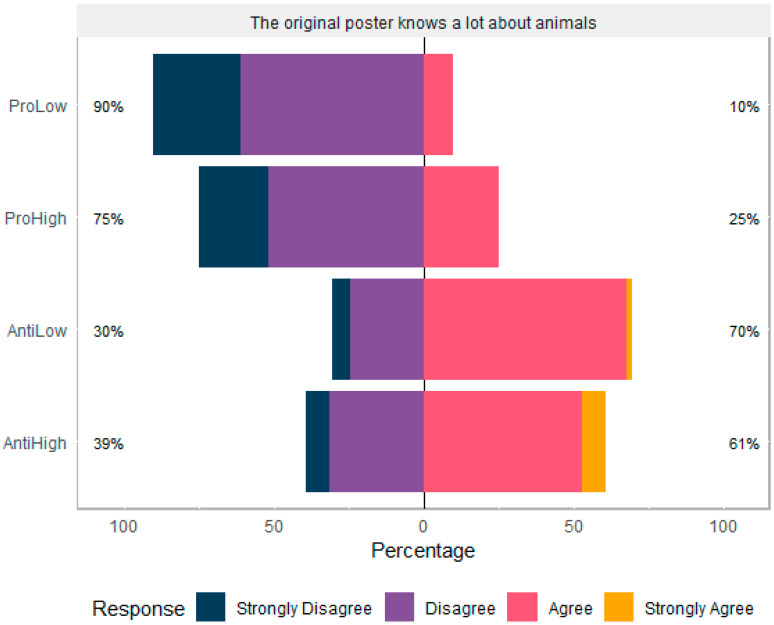
Participants’ (N = 211) agreement with the statement “The original poster knows a lot about animals”.

There was no effect of gender, education, or age on participants’ rating of poster’s knowledge. However, the anti narrative SMS context was rated as more knowledgeable about animals (H = 52.584, df = 3, *p* < 0.001, Figure 3).

### 3.3. Participants’ Attitudes to Commenters

#### 3.3.1. Comments Comparing Primates to Domesticated Dogs

**Figure 4:**
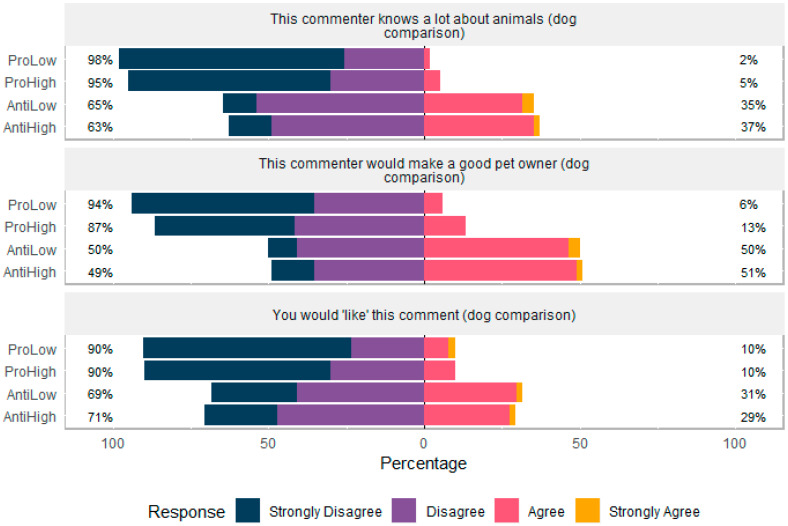
Participants’ (N = 211) agreement with commenter statements comparing primates to domesticated dogs.

Participants viewed a statement comparing primates to domesticated dogs and were asked if they agreed with three statements: “This person knows a lot about animals”, “This person would make a good pet owner” and “You would ‘like’ this comment”. There was no effect of age, gender or education on participant responses. The anti commenters were more often considered more knowledgeable (H = 78.119, df = 3, *p* <0.001) than the pro commenters. The anti commenter was more often considered a good pet owner (H = 58.943, df = 3, *p* < 0.001), and participants showed a higher tendency to hypothetically like the anti comment (H = 32.049, df = 3, *p* < 0.001, Figure 4).

#### 3.3.2. Comments Considering Domesticated Primates

**Figure 5:**
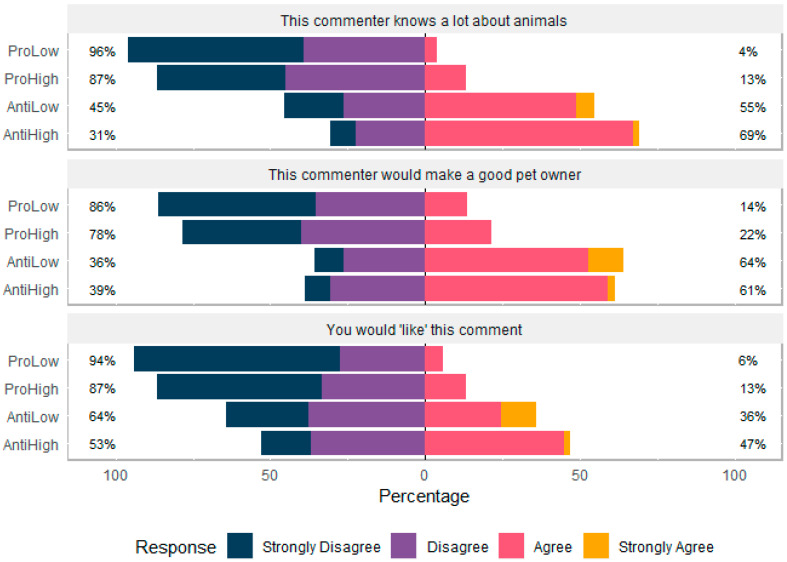
Participants’ (N = 211) agreement with commenter statements discussing primates as pets.

When shown the discussion of whether primates constitute a domesticated species, the anti comment was more often considered knowledgeable (H = 66.668, df = 3, *p* <0.001) than the pro comment. The anti commenter was more often considered a good pet owner (H = 55.76, df = 3, *p* < 0.001) than the pro commenter. Finally, participants were more likely to say they would ‘like’ the anti comment than the pro comment (H = 43.638, df = 3, *p* <0.001, Figure 5).

### 3.4. Free Text Responses

The themes identified in the free text responses are characterized in Table 4.

Some participants said they would not participate in the discussion, but others admitted they may respond in a certain way while privately holding other opinions.

*“I wrote a nice message on the facebook page, but I really think it would be silly to get a monkey. They are not domesticated animals!” *

*-Pro Narrative*

*“Normally, I wouldn’t post any comment on the page but since the survey required me to, I was being optimistic for both the owner and the monkey wishing them good fortune because from the comments I saw, I would have felt bad posting the only negative comment.” *

*-Pro Narrative*

We termed a common theme ‘reservations’. These comments often asked the original commenter a question which was designed to encourage critical thought about having a monkey as a pet, while not attacking the original commenter directly. They used language to soften their comment, often starting with a positive statement and then asking questions to encourage the poster to think critically, or expressing reserved doubts about the practice.

*“Good luck taking care of it. From what I’ve heard they’re more difficult to take care of than a human baby.” *

*-Pro Narrative*

*“So cute! Are you sure that it would make a good pet, though?” *

*-Pro Narrative*

*“Adorable! I’m not so sure a monkey’s place is in a human home, though.” *

*-Pro Narrative*

This theme was contrasted with ‘aggression to poster’ where the participant left a response which could be considered openly hostile, attacking the commenter’s beliefs or attempting to provoke a response.

*“You are an idiot.” *

*-Pro Narrative*

*“Do they taste delicious?” *

*-Anti Narrative*

Interestingly, there was no significant difference in the number of commenters who responded aggressively between contexts, but ‘reservation’ was more commonly observed in the pro narrative participants (χ2 (1, N = 211) = 15.9, *p* < 0.001, OR = 12). There was also no significant differences between age groups and their likelihood to respond with reservations.

Unsurprisingly, stating the cuteness of the monkey was more common in the pro narrative group (χ2 (1, N = 211) = 11.53, *p* < 0.001, OR = 7). Cuteness, however, could be considered independently of the primate’s ‘pet’ status.

*“Adorable! I wish I could have one.” *

*-Pro Narrative*

*“Very cute but beware because it is still a wild animal and its actions are unpredictable.” *

*-Pro Narrative*

Across both the narratives, there were comments which were concerned about the level of care the primate would require. There was no significant difference in the proportions of comments across contexts, but there was often a connection between this theme and the idea of ‘reservation’, with participants querying how the primate would be cared for.

*“Are you equipped to care for him? Is your house safe for him? Is where you’re living similar to where he’s from? Can he survive outside of his normal habitat?” *

*-Pro Narrative*

*“If properly cared for, monkeys can make great pets!” *

*-Anti Narrative*

There were also participants who explicitly considered the keeping of primates to be dangerous, either to the owner or the public.

*“You will never be able to control a wild animal.”*

*-Anti Narrative*

*“Scary”*

*-Pro Narrative*

Curiously, participants who liked the idea of monkeys as a pet appeared to respond differently based on context. Across both narratives, eight participants responded they would like a monkey as a pet, and there was no difference in proportion across narratives. However, within the anti context only, there was a style of comment defending the practice of keeping monkeys generally, while not expressing a personal desire to keep monkeys. This type of theme was not expressed by participants in the pro narrative.

*“Responsible owners can raise exotics pets, yes most people would not be capable but that doesn’t mean everyone” *

*-Anti Narrative*

*“People have had monkeys as pets for years, never really been an issue. Why now?” *

*-Anti Narrative*

## 4. Discussion

### 4.1. The Effect of Social Media on Animal Welfare Attitudes

This study had two main hypotheses: that participants exposed to a pro primate pet keeping Facebook group would have more favorable attitudes to primate pet keeping than those exposed to an anti primate pet keeping Facebook group and that participants exposed to posts with a high social loading would express stronger opinions than those exposed to posts with a low social loading.

In this study, the significant differences were mainly between the context of the narrative (pro vs anti). The social loading (high vs low) of the post was less important. Previous work has indicated that a desire for ‘likes’ and ‘shares’ (hereafter ‘engagements’) encourage sharing on social networks [10], and this behavior is strongly associated with a sharer’s narcissistic traits [39], which was not measured in the present study. It is presently unknown how engagements influence users’ knowledge-gathering behavior. SMS users often underestimate how many people would see their information [40] so it is possible that users may not recognize that a high engagement post means more people have seen the content.

The pro/anti narrative affected how the participants rated the primate’s emotional state. While participants were not able to freely choose emotional states and there was no ‘opposite’ emotion to ‘stressed’, e.g., ‘relaxed’, we can interpret this result with caution. Participants exposed to the pro narrative were almost three times more likely to agree the primate was stressed. This indicates that participants’ beliefs about the primate’s welfare were very much affected by the editorial information on the page. This fits with previous work which explored how participants rated the moods of chimpanzees and found that chimpanzees pictured with humans were rated as being more stressed or scared [15]. However, the results of the present study did not demonstrate that a pro narrative made participants more likely to want a primate as a pet.

The commentary and social response statements showed significant differences between the pro and anti narratives, with statements containing anti primate pet keeping sentiments consistently being rated as more knowledgeable about animals and coming from better pet owners.

Framing theory suggests that most attitudes are weakly held and easily influenced by contextual information and contrasting frames often lead to statistically significant effects [22,41]. It is worth noting that contrasting frames are not unrealistic nor unimportant to study, e.g., positive framings result in more egalitarian allocation of funds [42]. It’s thought that frames can become embedded in a society and define an issue, affecting how reality is perceived [43]. However, the link between attitudes and behaviors is not always clear [44]. A common example is the theory of planned behavior change, commonly used in public health, which suggests attitudes affect an individual’s perceived control of their behavior, but its utility in affecting public health changes is debated [45,46]. This has also been observed in social media studies, e.g., the framing surrounding the 2013 Singapore protests was successful in mobilising protests only in the short term and did not lead to sustained movements [47]. If attitudes to primates are weakly held and easily influenced by frames, can they affect animal welfare?

### 4.2. Engaging in Animal Welfare Debates

In their responses to the discussion of the social network, participants were overall more likely to be critical of keeping wild primates captive. However the qualitative comments revealed that a user’s behavior may not always reflect their beliefs. Homogenous clusters form in social networks [48] colloquially referred to as ‘echo chambers’, where the same opinions are expressed repeatedly. In this study we showed how echo chambers may begin to form as participants elected not to respond or to mask their true feelings. The ‘reservations’ theme is a demonstration of this. Instead of agreeing with the original post and the fictional commenters, these participants suggest a new perspective, but in a tone intended to be constructive. The participants saw a static set of comments, but in a real social network those participants’ comments would have been seen by other users, further reinforcing the echo chamber. There are many possibilities for exploring this in future work. For example, using a social identity theory lens may explain why participants are reluctant to disagree, out of concern of finding themselves part of an ‘out’ group [49]. Other theories of culture and identity may offer alternative, useful explanations for why this behavior exists and what its implications are for HAI.

One study of a particularly memetic video [19] found that as understanding of conservation issues entered the public narrative, significantly fewer commenters expressed a desire to keep a slow loris as a pet. During the same period, the proportion of references to the illegality of trade or painful procedures remained the same. They also highlighted that some commenters on the video considered the video to raise awareness of these conditions, although the trends in the comments did not necessarily support this. By contrast, in this study several participants within the anti narrative were driven to defend the practice of keeping monkeys without expressing a desire to keep one themselves. The content of participants’ responses did not always reflect the attitudes we observed in the quantitative aspect of the study. This is similar to the finding that Facebook users changed the words they used when presented with more content of a certain emotional context [50]. In that study, participants who were exposed to content with a negative emotional valence began to use more negative wording. The study was heavily criticized for manipulating the feeds of Facebook users without their knowledge and reflects the evolving nature of research ethics in these digital spaces, which is a topic of heated debate [51,52,53]. Our study deliberately chose to recruit participants to a scenario which was obviously a study, instead of creating fake Facebook pages and observing real-world behavior, as a result of ethical concerns, but this work suggests that it may be worth exploring a larger dataset collected from real world data to see if these effects persist outside of an experimental environment, and indeed what behaviors are affected by attitudes. If so, SMS platforms may need to do more to police content on their sites which may affect animal welfare. In late 2017, the influential site Instagram, owned by Facebook, implemented a tone policing policy for wildlife trade [54], where hashtags associated with animal abuse or wildlife trade will alert the user that animal exploitation is against Instagram’s Terms of Service. It is not yet known how impactful such interventions are, although ‘nudging’ through interface design is considered a potential avenue for behavioral change [55]. At present, Facebook’s moderation policy is ‘upon report’, not using policed hashtags. A 2017 leak of Facebook training material suggested that Facebook actively allowed imagery of animal abuse restricting only cases of sadism and celebration [56]; however, Facebook’s policy on content policing remains highly controversial, with inaccurate or damaging content only being grounds for review, not a breach of terms of service [57]. Given the high profile ‘fake news’ scandals (see [58]), content around animal welfare may not be addressed for some time, and the difficulty of implementing such an alert system will be challenging for operators.

### 4.3. Limitations

There are a number of limitations to this study. The relatively small sample size means all findings should be interpreted with caution and may not be generalizable to larger populations. Often studies of SMS are conducted on thousands of individuals in situ, allowing for more confidence in effect size [47,59]. However, as we were conducting an early study into an unknown area, we wanted to ensure participants were willing and fully informed regarding the work, as per the Association of Internet Researcher guidelines [32]. For this reason, we designed the survey with the mock site, although it clearly cannot fully replicate the SMS experience, and the charity incentive may have biased some participants. We consider that the survey format and our findings create a baseline for further work and perhaps justification for in-situ studies. There are a number of factors still to be considered, for example whether gender influences attitudes to animal welfare [60] and mediates SMS usage [61]. While there was no effect of gender observed in this study, a larger sample may find otherwise. In addition, while there was no observed effect of educational status in this study, previous research has shown that exposure to animal related courses influences attitudes to animals [62]. Further work should also explore past animal experiences, including experiences with companion animals, and their influences on these behaviors. As previously mentioned, we chose to use an even Likert scale to force choices, and we did not present a neutral framing of the primate to observe how participants responded to the image without bias. The role of ambiguity in this arena of study is still unclear and could also be explored.

## 5. Conclusions

The present study builds on a body of work exploring how specific platforms may ‘tone police’ animal welfare challenges within their community.

This study found some limited evidence that the content of SMS can moderate attitudes to animal welfare issues, particularly in how users might respond in line with an existing community’s norms. The most important finding of this study is its implication that the expressed belief may not be the true belief, which was most clearly demonstrated within the qualitative data. We suggest future studies of HAI consider the specifics of digital culture research to understand how HAI are represented and codified and the impacts this may have on both human and non-human agents. This may be more important than ever in a post-COVID-19 world, where whole populations are using SMS as a primary communication tool. This study has demonstrated that the context of these SMS communications influences human attitudes and potentially their behavior in response to the sharer, and so future studies must be aware of the human-to-human influences in HAI studies, perhaps especially in virtual spaces.

## Figures and Tables

**Table 1:** Respondent demographics across condition, N = 211.

Condition	Anti Primate Keeping High Social Loading	Anti Primate Keeping Low Social Loading	Pro Primate Keeping High Social Loading	Pro Primate Keeping Low Social Loading
**Gender**				
Male	11.4% (N = 24)	14.3% (N = 30)	13.8% (N = 31)	8.1% (N = 17)
Female	11.4% (N = 24)	9.5% (N = 20)	13.8% (N = 29)	14.8% (N = 31)
In another way *	0.5% (N = 1)	1.0% (N = 2)	0	0.5% (N = 1)
**Age**				
18–29	12.9% (N = 26)	15.4% (N = 31)	15.4% (N = 31)	12.9% (N = 26)
30–44	10.9% (N = 22)	8.9% (N = 18)	12.9% (N = 26)	10.9% (N = 22)
**Education Status**				
Some college or less	10.9% (N = 23)	10.9% (N = 23)	13.3% (N = 28)	10.4% (N = 15)
Bachelor Degree	8.5% (N = 18)	7.1% (N = 15)	10.9% (N = 23)	10.4% (N = 22)
Masters or higher	3.8% (N = 8)	6.6% (N = 14)	4.3% (N = 9)	6.2% (N = 13)

* In gender breakdowns ‘in another way’ was not included.

**Table 2:** Pro and anti narrative commentary and social network response comparison.

Theme	Text
Pro Exotic Pet Keeping	
Original Poster’s Commentary	Cute! My baby is ready to come home from the breeder!! Now that all of my kids are gone, I am so lucky to welcome my new MONKID to the family.
Response Comment Theme: Human Comparison	Aw! Just like a baby!
Response Comment Theme: Comparison with Domestic Animal	Ugh! I want one!! I already have a dog … how hard could it be? They aren’t that different
Response Comment Theme: Suitability of Pet	I bet this guy makes a perfect pet. So many snuggles! Plus exotics bond to their owners & enjoy living w them!!
Anti Exotic Pet Keeping	
Original Poster’s Commentary	These breeders should be ashamed. Monkeys belong in the wild and not in homes as pets!
Response Comment Theme: Human Comparison	Stop treating them like babies
Response Comment Theme: Comparison with Domestic Animal	Exotic animals aren’t like dogs. They don’t make good pets. Zoos can’t properly take care of them, how could a private owner?
Response Comment Theme: Suitability of Pet	Wild animals will never be domesticated and are rarely tame. Keeping them at home will be damaging. To them and the owner.

**Table 3:** Survey questions used for all treatments and their response types. *Name would be “Animal Freedom” for negative posts, “Monkey Babies” for positive posts.

Number	Question	Response Type
1	If you were to respond to this discussion, write your response below.	Open Response
2	The environment you saw in the picture was appropriate for that animal.	4-Point Likert Scale *
3	This animal will make a suitable pet.	4-Point Likert Scale *
4A	Would you like this animal as a pet?	Yes/No
4B	Other comments?	Open Response
5	How do you think this animal feels?	Multiple Response
	Choices (nonexclusive)	
	Happy	
	Sad	
	Excited	
	Stressed	
	Don’t Know	
6	The page * [name] is knowledgeable about animals.	4-Point Likert Scale *

* Levels: Strongly Disagree, Disagree, Agree, Strongly Agree.

**Table 4:** Themes identified from participants’ responses to the Facebook discussion and differences between pro and anti primate pet keeping conditions.

Theme	% of Comments in Anti Context (N)	% of Comments in Pro Context (N)	χ^2^ (Fisher’s Exact Test True Odds Ratio ≠ 0 *p*; 95% CI)	Example Comment
Active Opt Out	5.9% (N = 6)	3.6% (N = 4)	0.62 (*p* = 0.52; 0.12, 2.61)	I would totally_never_respond to this discussion.
Aggression to Poster	4.0% (N = 4)	4.6% (N = 5)	0.44 (*p* = 1; 0.24, 5.99)	This is disgusting! Wild animals are NOT pets. They belong in the wild!
Monkey is Cute	3.0% (N = 3)	17.3% (N = 19)	11.54 (*p* < 0.001; 1.90, 36.89)	Monkeys are the cutest!
Monkey is Dangerous	7.9% (N = 8)	2.7% (N = 3)	1.92 (*p* = 0.123; 0.05,1.42)	Too many accidents can happen when keeping wild animals in your home.
Legal Doubts	1.0% (N = 1)	3.6% (N = 4)	1.59 (*p* = 0.371; 0.36, 187.49)	Adorable! Are monkeys allowed as pets in the US?
Monkeys Can Be Pets	9.9% (N = 10)	0	9.35 (*p* < 0.001; 0, 0.38)	If properly cared for, monkeys can make great pets!
Reservations	2.0% (N = 2)	19.1% (N = 21)	15.87 (*p* < 0.001; 2.70, 104.68)	Is the home really a better place for monkeys than the wild?
Wild Animals Should Be Free	28.7% (N = 29)	15.5% (N = 17)	5.43 (*p* = 0.03; 0.22,0.93)	This is a wild animal and should not be contained in a cage. It has special needs and requirements that a normal person can not give it.
Wild Animals Require A Lot of Care	14.9% (N = 15)	9.1% (N = 10)	1.67 (*p* = 0.209; 0.22, 1.45)	Owning a monkey seems like a huge responsibility.
I Would Like a Monkey	1.0% (N = 1)	6.4% (N = 7)	4.17 (*p* = 0.067; 0.84, 308.85)	I would like one but i have 3 dogs allready hands full
